# Coexistence of adenomyomatosis in a left-sided gallbladder: a case report

**DOI:** 10.1186/s12245-024-00785-0

**Published:** 2025-01-03

**Authors:** Mohamed Tolba, Hadeer Hafez, John Adel

**Affiliations:** 1 Faculty of medicine, Misr university for science and technology, Giza, Egypt; 2 Faculty of Medicine, October 6th University, Giza, Egypt; 3 Department of general surgry, Faculty of medicine, Misr university for science and technology, Giza, Egypt

**Keywords:** Left-sided gallbladder, Adenomyomatosis, Congenital anomaly, Cholecystectomy, Hepatocellular carcinoma

## Abstract

**Introduction:**

The coexistence of gallbladder (LSG) and adenomyomatosis (ADM) is extremely uncommon presenting a novel clinical dilemma that has not been previously documented. LSG refers to a anomaly where the gallbladder is situated to the left of the round ligament deviating from its usual position. This anomaly is rare, with reported occurrences ranging between 0.04% and 1.1%. Identifying LSG before surgery poses challenges. It is often discovered incidentally during procedures necessitating surgical expertise to safely manage anatomical variations.

**Case presentation:**

We report an old man with a history of hepatitis C, carcinoma and liver cirrhosis complained of sudden epigastric pain. A CT scan revealed the presence of an LSG, which’s a congenital anomaly. During the cholecystectomy procedure surgeons encountered variations and observed the existence of ADM complicating the operation. The patient recovered smoothly post surgery.

**Discussion:**

This case shows how complicated it can be to diagnose and treat the combination of LSG and ADM. Identifying these conditions before surgery is tough so surgeons often have to adjust their approach during the operation. Although laparoscopic cholecystectomy for LSG is usually safe it requires care to avoid problems like bile duct injuries. For patients at risk a conservative treatment approach might be better. In cases where surgery is necessary surgeons need to adapt their techniques to address the unique anatomical issues.

**Conclusion:**

The combination of LSG and ADM in a setting poses an intricate challenge. Surgeons need to be ready to recognize and address these abnormalities effectively for the well being of the patient and favorable results. This particular case highlights the importance of staying alert and flexible during surgery when dealing with gallbladder variations.

## Introduction

Left sided gallbladder (LSG) and adenomyomatosis (ADM) coexistence is very rare and it leads to a special medical dilemma that has never been reported before in literature. While cholecystitis ranks among the most frequently encountered surgical cases in the medical field, the presence of a sinistropositioned gallbladder or left-sided gallbladder is an uncommon occurrence [1]. Typically, the gallbladder is situated in the gallbladder fossa, nested between hepatic segments IV and V, LSG diverges from cases of the gallbladders conventional placement. LSG, characterized by the gallbladders left sided position relative to the round ligament or ligamentum teres (the thickened, free edge of the falciform ligament of the liver), represents a rare congenital anomaly. Its reported occurrence varies between 0.04% and 1.1% [1, 2]. Most of the reported instances are linked to a falciform ligament on the right side, and they are referred to as a false left-sided gallbladder. In cases where the falciform ligament is not positioned on the right side, it is termed a true LSG, an extremely uncommon occurrence [3]. This infrequent discovery often happenstance during laparoscopic procedures, as pre-operative evaluations might overlook this anomaly [3]. Variations in anatomical structure can introduce surgical complexities and heightened morbidity risks. The ability to identify such anatomical deviations is an essential surgical skill for ensuring procedural safety [4]. Prior studies have highlighted instances of LSG occurring in diverse locations [5–8]. To our best knowledge, the simultaneous presence of LSG and ADM has not been documented thus far. In this case report we discuss the Presence of these two conditions, highlighting complexity of diagnosis, surgical treatment and seeking the best patient care.

## Case presentation

A patient in his 60s presented with acute and persistent epigastric pain. Accompanied by nausea and vomiting. The patient medical history revealed a pre-existing, neglected, case of hepatitis C virus (HCV) infection Which had progressed to hepatocellular carcinoma (HCC) over time. Furthermore, the patient exhibited liver cirrhosis, with compensated hepatic cell failure, which was compounded by the presence of mild ascites. Associated with malignant thrombus present in the IVC extending to the right atrium and another thrombus of the portal vein.

physical examination showed guarding upon palpation of the epigastrium, mass in the same region, and positive Murphy sign. accompanied by a low-grade fever (37.9 C) Notably, the presence of caput medusa on the abdominal region was also recognized indicating extensive portocaval anastomosis.

Laboratory investigations were conducted, and their findings were elevated leucocyte count ( 14*10^3^/UL), normal liver functions WBC’s 14*10^3^/UL (*serum direct billirubin:0.37 mg/dl*,* T. Bilirubin: 0.8 mg/dl)*, Normal kidney function *(Blood urea nitrogen (BUN)) = 72 U/l*,* Elevated C-reactive protein (CRP) = 65*,* Gamma-Glutamyl Transpeptidase (GGT) = 233 mg/dl*,* Alkaline Phosphatase (ALP) = 167 mg/dl*, serving as a crucial reference for the diagnostic process. The patient was admitted to the surgical ward for monitoring and further investigations. Intravenous administration of antibiotics, coupled with the provision of supportive fluids and medications, was promptly initiated. An abdominal computed tomography (CT) scan and laboratory investigation was conducted. After 48 h, comprehensive laboratory were done which revealed that WBCs:18.1*10^*3*^*UL Serum direct bilirubin:0.76 mg/dl*,* Total bilirubin:1.82 mg/dl*,* BUN:49U/l*,* CRP:50*,*GGT:191 mg/dl*,* ALP:217 mg/dl.* highlighting a concerning decline in the patients overall clinical status.

**Fig. 1:**
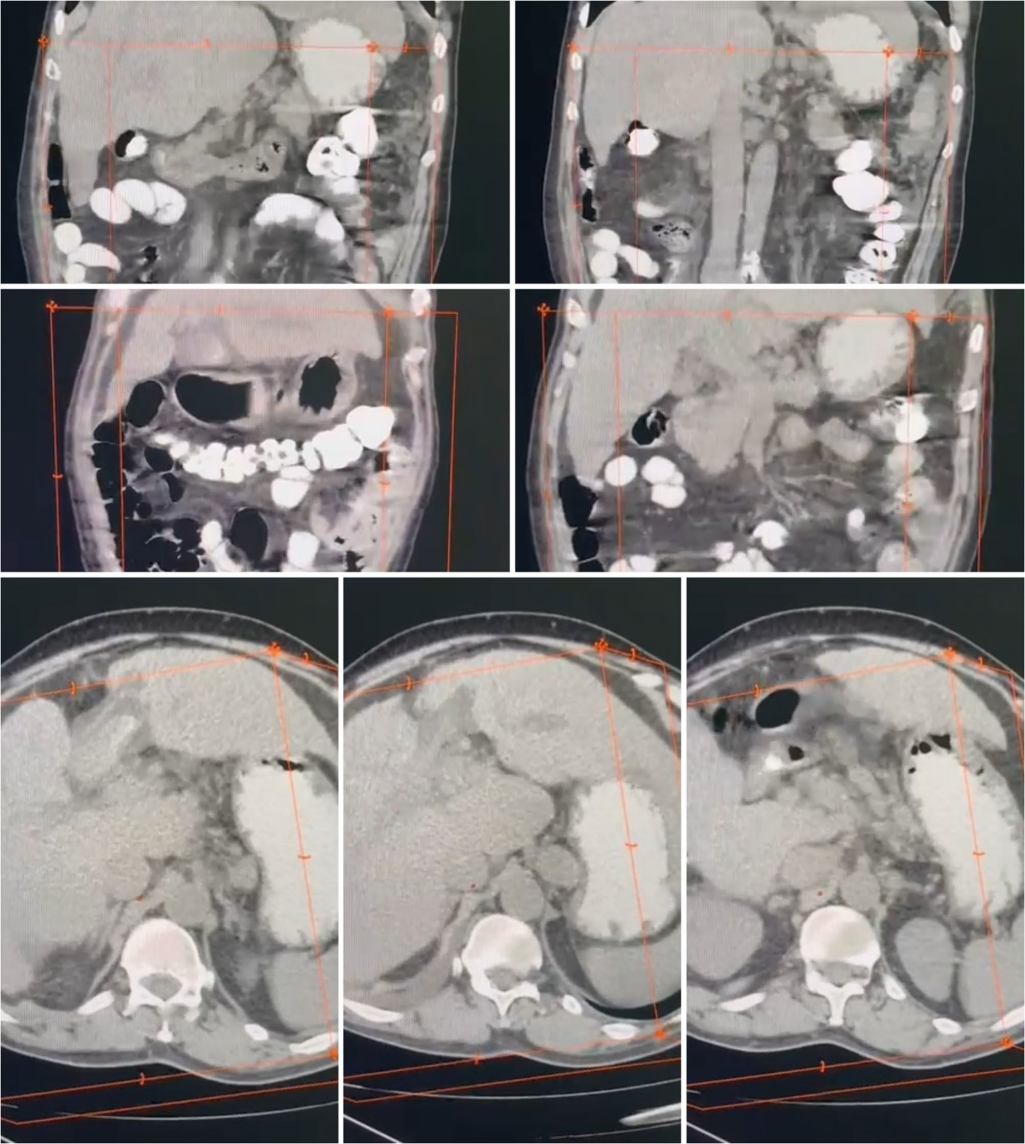
The computed tomography (CT) Cuts delineated an anatomical positioning of the gallbladder, indicative of a true left-sided configuration

The computed tomography (CT) report delineated an anatomical positioning of the gallbladder, indicative of a true left-sided configuration, concomitant with indications suggestive of acute cholecystitis (Fig. 1).

**Fig. 2:**
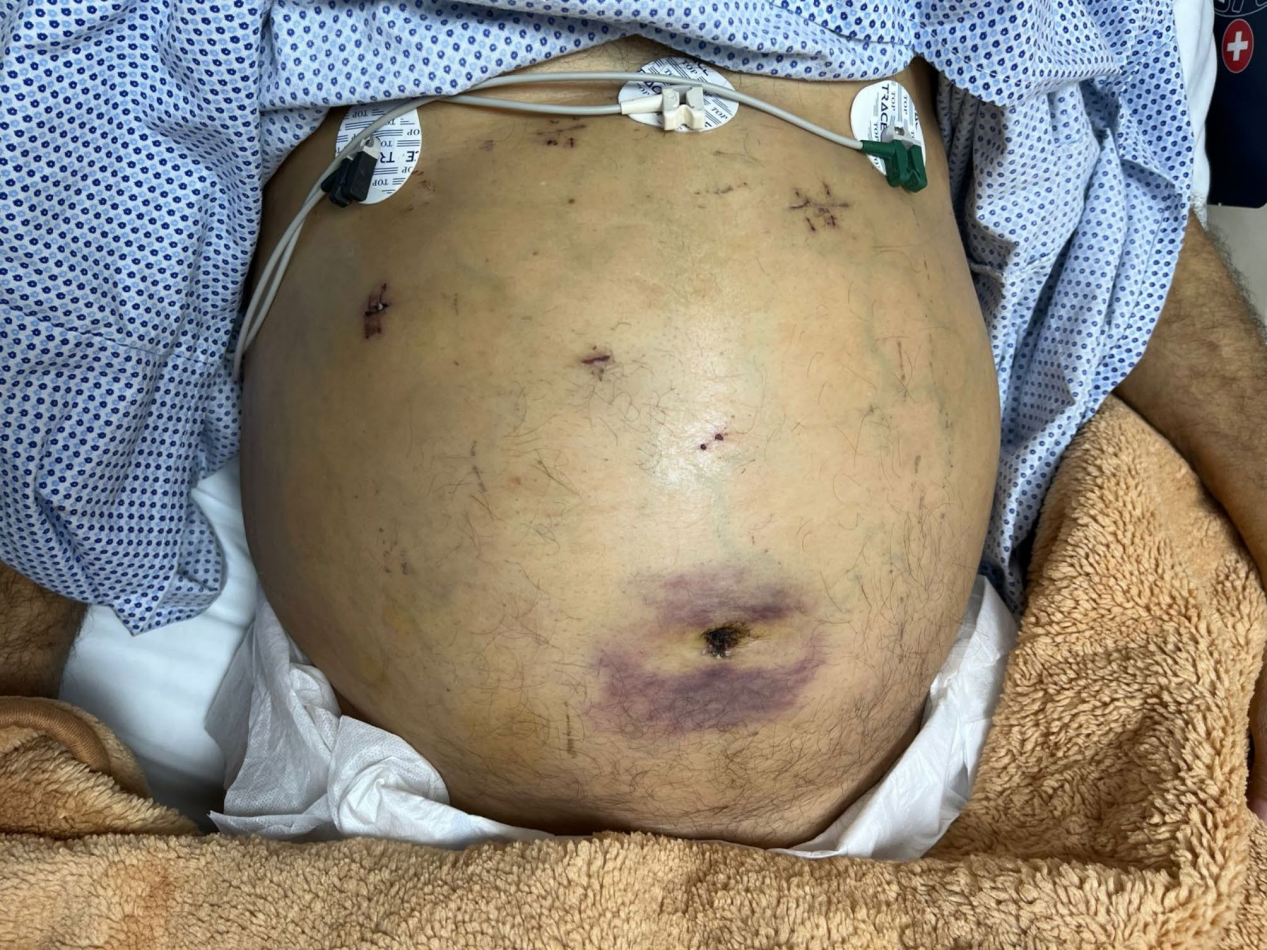
Shows modified ports positions to accommodate patient special anomaly

The Criticality of the patient condition, his rapidly declining clinical status, and the failure of conventional supportive interventions were all factors that led the attending surgeon to speed up the surgery to be done within 48 h of admission. Also due to the unique anatomical constraints, a paramount consideration was taken to avoid compromise of the distended caput medusae. the surgeon modified the positioning of the laparoscopic port incisions to enhance accessibility to the targeted organ (Fig. 2).

**Fig. 3:**
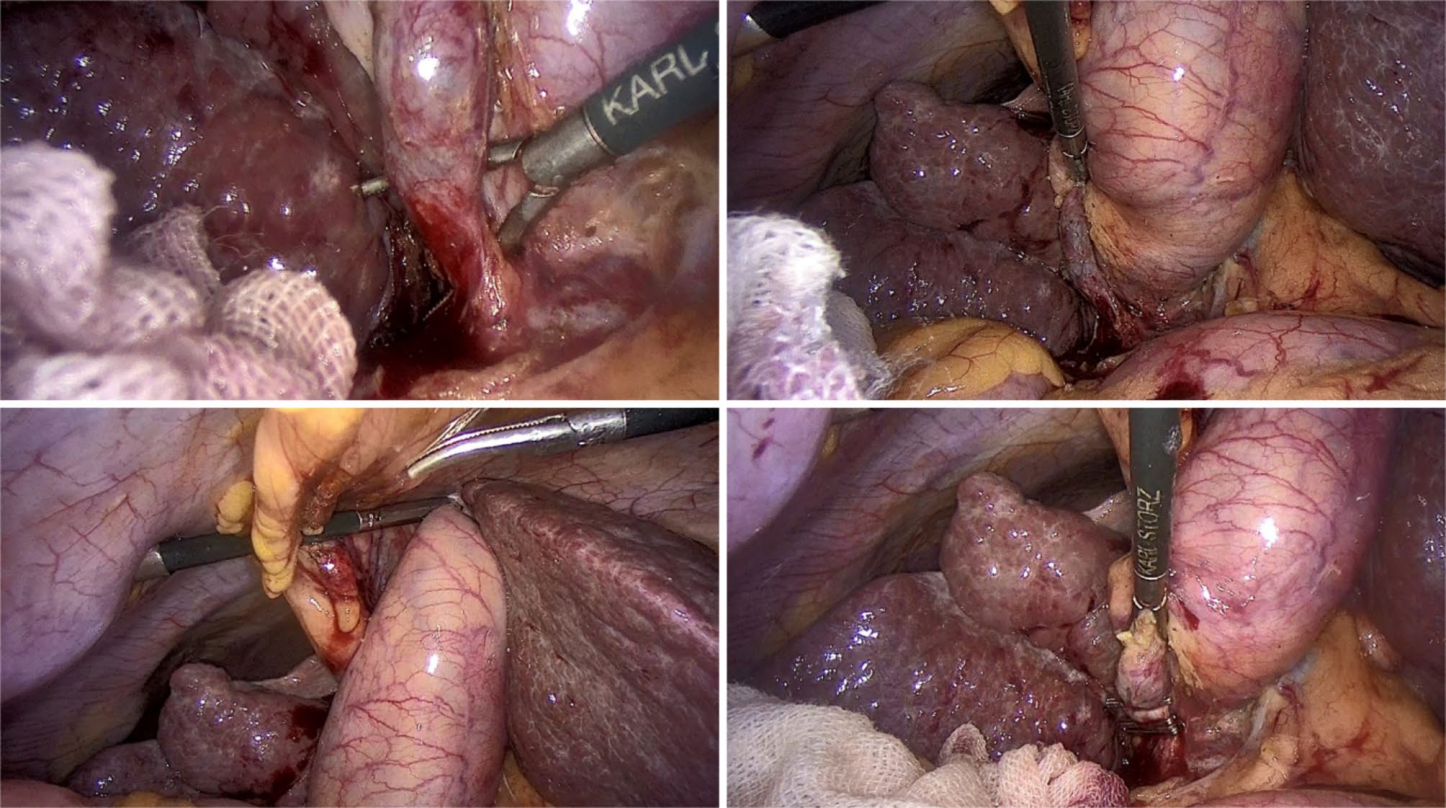
Calots triangle was incorrectly positioned, with the cystic artery amid the cystic duct and common bile duct

During visualization, signs of acute inflammation were noted, along with omental adhesion requiring dissection and ascites aspiration. Upon gallbladder descent, Calots triangle was incorrectly positioned, with the cystic artery amid the cystic duct and common bile duct (Fig. 3). This matched the CT report and diagnosis of sinsitropositioned gallbladder. After gallbladder extraction, an unusual mass was felt within its wall, where sample dissection revealed adenomyomatosis that was confirmed by the pathologist. Postoperatively there was no complications.

The patient was discharged from the hospital after an observation period of three days, with subsequent scheduled appointments planned for comprehensive follow-up evaluation after one week. On follow up visit, patient showed good general condition with improved blood work up. Suddenly, patient admitted to the hospital ICU due deterioration in liver function due to causes that are not related to the procedure.

## Discussion

This surgical case report discusses the exceptionally rare coexistence of a LSG and ADM, presenting a unique medical dilemma not previously reported. While cholecystitis is a common surgical condition, the infrequent occurrence of LSG, particularly in the absence of a right-sided falciform ligament, adds complexity to pre-operative evaluation and surgery plans. Detection of these anomalies is often incidental during laparoscopic procedures, highlighting the need for surgeons to be adept in recognizing such variations. While laparoscopic cholecystectomy for a left-sided gallbladder is considered safe, it carries an increased risk of complications, notably common bile duct injuries [9, 10]. As a result, surgeons are recommended to exercise greater caution during the procedure. This caution includes minimizing the use of diathermy and taking meticulous care when dividing structures to prevent potential intraoperative injuries [11]. Diagnosing this case was challenging due to signs and inconclusive initial ultrasound results. And usually, these two conditions do not appear in imaging diagnostic methods. Despite the patients condition. surgical intervention was necessary due to the emergent nature of the condition; Even with additional health issues, like hepatitis C virus infection and hepatic cellular carcinoma, as this normally prohibits surgical intervention. In other case report, the LSG was discovered when Magnetic resonance imaging (MRI) showed that the gallbladder was situated to the left of the fissure for ligamentum teres, beneath segment III [10]. In another case the anomalous anatomical position of the gallbladder was only identified during the operative course [11]. Due to a preoperative misdiagnosis of LSG, surgeons in a similar case proceeded to perform a laparoscopic cholecystectomy on the patient in the French position, utilizing the standard placement of four ports. Upon discovering that the gallbladder was not in its expected location, the surgical team subsequently conducted a retrograde cholecystectomy using electrocautery, followed by an intraoperative cholangiogram [3].

During the operation the surgeon had to adapt his approach to accommodate the challenges posed by the left sided gallbladder. Modifications were made to the procedure considering adhesions and unexpected anatomy that added complexity to the surgery. Furthermore, discovering ADM within the gallbladder further complicated the process.

For patients with acute gallbladder issues who have additional comorbidities, it’s wise to consider a conservative approach rather than immediate surgery. This helps reduce the chances of further issues. When surgery is unavoidable, the surgeons should be experienced in changing the surgical incisions to match the patient’s unique anatomy, as variations can create challenges, especially in cases involving a caput medusae. Surgeons need to be careful and accurate when placing the incisions to handle these variations effectively. By following these recommendations, we can improve patient care and outcomes when dealing with the variations in gallbladder anatomy in acute cases.

## Conclusion

This case scenario underscores the importance of recognizing differences like LSG and ADM when planning out surgical procedures. It is crucial to identify these variations and modify surgical methods to effectively address these anomalies. Thereby, protectig patient well being and achieving favorable results.

## Data Availability

No datasets were generated or analysed during the current study.
